# A synthetic peptide as an allosteric inhibitor of human arginase I and II

**DOI:** 10.1007/s11033-021-06176-5

**Published:** 2021-02-15

**Authors:** Kai Gao, Sergey Lunev, Mariska P. M. van den Berg, Zayana M. Al-Dahmani, Stephen Evans, Dyon A. L. J. Mertens, Herman Meurs, Reinoud Gosens, Matthew R. Groves

**Affiliations:** 1 Structure Biology, Drug Design Group Departments of Pharmacy, University of Groningen, Groningen, The Netherlands; 2 Immunopharmacology, Department Molecular Pharmacology, University of Groningen, Groningen, The Netherlands; 3 Molecular Pharmacology, Department of Pharmacy, University of Groningen, Groningen, The Netherlands; 4 EV Biotech, Groningen, The Netherlands

**Keywords:** Arginases, Urea cycle, Peptide analogue, Inhibitor, Microscale Thermophoresis, Colorimetric measurement

## Abstract

Arginine metabolism mediated by arginases plays a critical role in cell and tissue function. The arginine hydrolysis is deeply involved in the urea cycle, which helps the kidney excrete ammonia from blood. Upregulation of arginases affects microenvironment stability due to the presence of excess urea in blood. To regulate the arginase activities properly, a synthetic peptide based on the structure of human arginase I was designed and assessed. Preliminary data shows it inhibits human arginase I and II with an IC_50_ of 2.4 ± 0.3 and 1.8 ± 0.1 mmol, respectively. Our kinetic analysis indicates the inhibition is not competitive with substrate – suggesting an allosteric mechanism. This result provides a step towards specific inhibitors design.

**Supplementary Information:**

The online version contains supplementary material available at 10.1007/s11033-021-06176-5.

## Introduction

Arginases have been under investigation for over a century. These manganese-containing metalloproteins catalyze the formation of urea and L-ornithine from L-arginine, thereby serving various homeostatic goals. Firstly, arginases eliminate ammonia from the body through urea synthesis [1]. Secondly, L-ornithine serves as precursor for the production of polyamines and proline, which are essential for cell proliferation and collagen production [2, 3]. Finally, arginases also regulate the production of nitric oxide (NO) by competing in various organs with NO synthases (NOS) for their shared substrate L-arginine [4]. In mammals two isoenzymes of arginase occur: arginase I and II. Although similar in their function, the two isoforms differ in their subcellular compartmentation and genetic origin. Arginase I is a cytosolic enzyme that is highly expressed in the liver, whereas arginase II is found in mitochondria and is the dominant isoform in the kidney [1, 5]. However, both arginase I and II can be found in many different tissue and cell types throughout the body [1, 5, 6], and are found to be involved in the pathophysiology of various diseases; including pulmonary disease, atherosclerosis, cancer and vascular disease [2, 6–8].

The functions of arginases including metabolism, distribution, kinetic properties, quaternary structures, evolutional aspects and role in diseases have been extensively studied [9]. Significant amount of efforts have been focused on the highly conserved active site of arginases, which has led to the design of inhibitors in order to address the enzymatic activity by competing with substrate L-arginine. Subsequently, this strategy has evolved numbers of promising inhibitors with strong binding affinity to arginases: the hydroxy derivative of L-arginine resulted in L-N-hydroxyarginine (NOHA) [10], boronic acid modification of the side chain resulted in 2(S)-amino-6-boronohexanoic acid (ABH) [11] and S-(2-boronoethyl)-L-cysteine (BEC) [12]. However, due to the poor pharmacokinetics profiles, all these inhibitor candidates have not been successfully forwarded to clinical settings. Until in late 2019, a small molecule INCB001158 (CB-1158) targeting arginases for cancer therapy was firstly moved into Phase I/II clinical trials [13]. Therefore, for clinical applications, a well-designed arginase inhibitor is still urgently needed in the present.

A promising avenue in arginase inhibitors research is a structural feature that shared by all mammalian arginases – the S-shaped motif at the C-terminal [14–16]. The functional significance of this motif is that it accounts for 54% of intersubunit contacts [17]. In Human Arginase I (hargI) the S-shaped motif is comprised by residues 304–322, where Arg308 is thought to play the major role, as it forms a salt bridge with Glu262. In rat liver arginase I, Arg308 also forms an intermonomer salt bridge with Asp204 from an adjacent subunit [17]. The importance of Arg308 is supported by the discovery that a truncated hargI terminating at Arg308 retained the trimeric assembly of hargI [14], while both human and rat Arginase I Arg308Ala mutants were monomers in solution [15, 17]. Although the inter-oligomeric interactions were shown not to influence the catalytic behavior of hargI under optimal conditions (pH 9.5), at a physiological pH of 7.4 the Arg308Ala mutant did not show cooperative interaction with the substrate L-arginine, unlike its wild-type counterpart [17, 18]. Interestingly, the Arg308Ala mutant showed significantly increased activity as well as restored the trimeric assembly and substrate cooperativity in presence of low concentrations of guanidinium chloride (Gdn+) [17] – whereas the wild-type and truncated species terminating at either Arg308 or Ala308 were not responsive to Gdn + treatment even at higher concentrations. The truncated species were also not able to restore cooperativity to L-arginine [17]. Existence of such interactions can also be demonstrated by the crystal structure of *Bacillus caldovelox* arginase lacking the Arg308 homologous residue (construct terminated at Met299, PDB:3CEV), in which an L-arginine molecule was bound in the same inter-oligomeric interface region as seen for Arg308 in hargI [19]. This phenomenon raised our interest in the characterization of S-shaped motif at C terminal of hargI, as well as the key residue Arg308, since the motif tail influences the configuration of arginases I and activity of the enzymes, a peptidomimetic based on S-motif (residues 304–322) thus provides a rational route for arginase inhibitor design.

**Fig. 1:**
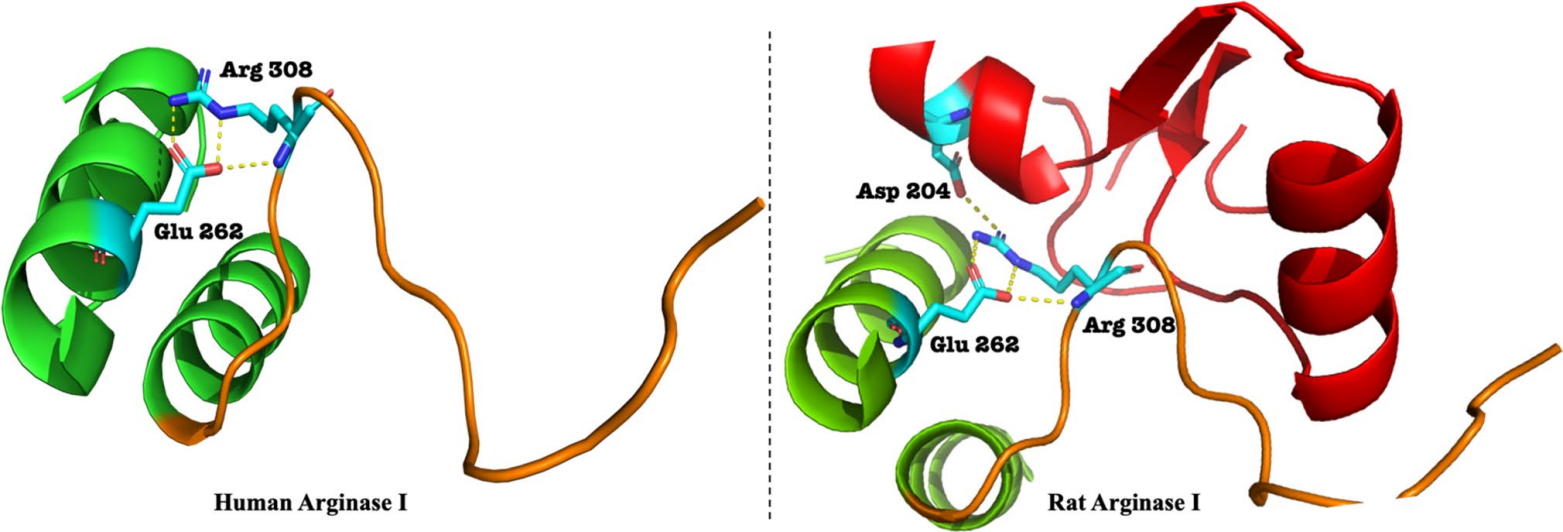
Arginase I structure of human (left, PDB: 4 IE1) and rat (right, PDB: 1RLA) showed a similar S shape tail (colored in orange) at the C terminal. The residue Arg308 forms 3 hydrogen bonds with Glu262 within the same chain in a dimeric form of human arginase I; For rat, besides the interaction with residue Glu262, Arg308 has an intermonomer interaction with Asp204 from adjacent subunit (colored in red), which help to form a homotrimeric structure of arginase I in rat

In this study, in order to test our hypothesis, we have designed an peptide analogue from the C-terminal region on hargI, and examined it in vitro effect upon the catalytic behavior. The synthetic peptide (REGNH) was assessed in an L-arginine metabolism assay against both hargI and hargII. Results in this report also further support the observation that hargI and hargII posse almost identical activities in vitro. Furthermore, the addition of peptide analogue in arginases mediated hydrolysis presents a non-competitive inhibition behavior against substrate, indicating its allosteric nature in this assay (Fig. 1).

## Materials and methods

### Protein sub-cloning, expression and purification

Cloning, expression and purification of human arginase I (hargI) were performed as described previously [21]. Briefly, the full-length gene encoding hargI was amplified using polymerase chain reaction (PCR) from a commercially available (dnasu.org) template plasmid using sequence-specific oligonucleotides containing BspHI and HindIII restriction sites and Phusion HF polymerase. The gene was further treated with BspHI and HindIII restriction enzymes and sub-cloned into pETM11 expression plasmid previously digested with NcoI and HindIII using T4 DNA-ligase. All amplification, digestion and ligation steps were performed according to the manufacturer’s recommendations (Thermo Scientific). The resulting plasmid pETM11-hargI encoded the full-length human arginase I with the additional His6-tag and TEV protease restriction site at the N-terminus as confirmed by Sanger sequencing.

The hargI was recombinantly expressed in *E. coli* BL21 (DE3) RIL cells (Novagen), cells were propagated in 0.5 L of selective autoinducing medium ZYM5052 in presence of 100 μg/mL kanamycin and 35 μg/mL chloramphenicol at 37 °C in a 2 L baffled Erlenmeyer flask (Nalgene, US) according to the autoinduction protocol [20]. After reaching an OD600 of approximately 2.0, the culture was cooled to 18 °C and followed by overnight expressing. The cell pellets were then harvested by centrifugation after 16 h. Cell pellet was further resuspended in 30 ml Lysis buffer [50 mM Tris-HCl pH 8.0, 300 mM NaCl, 20 mM imidazole, 5% (*v*/v) glycerol, 3 mM β-mercaptoethanol (BME)] supplied with 1 mg lysozyme (Alfa Aesar). The cells were lysed on ice by sonication (Branson W250D) and homogenate was clarified by centrifugation at 41,000 g for 60 min.

Soluble His6-tagged hargI was purified from supernatant by using 2 ml Ni-NTA Agarose (Macherey Nagel) in a gravity-flow column (Bio Rad) at room temperature. The Ni-NTA resin was further washed with 100 ml lysis buffer. Main protein was eluted with 10 ml of Elution buffer [50 mM Tris-HCl pH 8.0, 300 mM NaCl, 300 mM imidazole, 5% (*v*/v) glycerol, 3 mM BME]. The elution fraction was concentrated to 10 mg/ml and purified via a size-exclusion chromatography (SEC) using HiLoad 16/60 Superdex 75 column (GE Healthcare) equilibrated with SEC buffer (10 mM Ammonium acetate pH 7.3, 150 mM NaCl, 2 mM MnCl_2_, 5% *v*/v Glycerol, 3 mM BME) on the automated NGC chromatography system (BioRad). SEC buffer was chosen based upon a Differential Scanning Fluorimetry (DSF) assay [27]. The hargI was eluted as a single peak at an elution volume of approximately 70 ml. This peak was pooled, concentrated by using a centrifuge concentration unit (Sartorius) and stored in 50% (*v*/v) glycerol at −80 °C for further use. Final yield of purified hargI was approximately 40 mg per liter of bacterial culture.

The human arginase II (aa23-354, 38.3kD) fused to a His6-tag at the N-terminal was purchased and supplied as a liquid aliquot (20 mM Tris-HCl, pH 8.0, 10% (*v*/v) glycerol) at −20 °C (USBiological Life Science, Salem, USA). This stock was thaw on ice, followed by high speed centrifuging before diluting to certain concentration for activity assay.

The synthetic peptide REGNH resembling C-terminal region of hargI responsible for the inter-oligomeric interactions and substrate cooperativity was purchased in lyophilized form (Biomatik and TopPeptides, Shanghai) and aliquots were freshly dissolved in PBS buffer prior to experiments.

### Microscale thermophoresis (MST)

MST measurements were performed on a Monolith NT.115 instrument (Nanotemper Technologies, GmbH). Purified His6-tagged hargI sample was labeled with the Monolith His-Tag Labeling Kit RED-tris-NTA (MO-L008) according to the protocol (Nanotemper Technologies, GmbH). All measurements were performed in triplicate in MST buffer (25 mM Tris-HCl pH 7.5, 125 mM NaCl, 1% Glycerol and 1 mM BME, 0.05% Tween-20) using standard capillaries (K002, Nanotemper Technologies, GmbH). Labeled hargI was used at a final concentration of 50 nM according to the manufacturer’s recommendations. All measurements were performed at 60% LED excitation power and 40% MST power, Laser-On time was 30 s, Laser-Off time 5 s. The peptide REGNH was titrated in 1:1 dilution starting at 2.82 μM. All binding reactions were incubated for 10 min at room temperature followed by centrifugation at 20,000 g before loading into the capillaries.

### Enzymatic assay

The kinetic properties of hargI and II were tested according to previously reported methods [28–30] with minor modifications. The Arginases activity was assessed by colorimetric measurement of produced urea. The assays were performed in 96-well plates (Greiner) using Assay buffer [100 mM Na-phosphate pH 7.4, 130 mM NaCl]. Final volume of each reaction was 60 μl, the final concentration of hargI and hargII was 50 nM in each well. Samples containing no hargI or hargII were used as negative control. The peptide inhibitor was added based on experiment setting, dose ranging from 20 mM to 9.8 μM. Each sample was pre-incubated for 30 min in a 37 °C shaking incubator (100 rpm) and the reactions were started by adding concentrated substrate buffer [Assay buffer supplemented with 100 mM L-arginine, 500 mM Glycine and 1.25 mM MnCl_2_]. Reactions were performed in a shaking incubator at 37 °C and quenched after 1 h using a Stop mix. The Stop mix consisted of two volumes of freshly prepared solution A [Antipyrine/ H_2_SO_4_ reagent: 5 g/L of antipyrine(1,5-dimethyl-2-phenyl-3-pyrazolone) in 50%(*v*/v) sulfuric acid] and one volume solution B [Oxime reagent: 0.8 g 2,3-Butanedione monoxime in 100 ml of 5%(v/v) acetic acid]. After quenching, the reactions were allowed to develop color in the dark at room temperature for at least 18 h, followed by heating the mixtures at 45 °C in a water bath for 4 h. The color turns to brown and changes in optical density (OD) at 466 nm reflecting the production of urea were quantified using the Synergy H1 Hybrid Reader (BioTek).

## Results

Recombinant full-length Human Arginase I was cloned, recombinantly expressed in *E. coli* and purified to homogeneity. The use of the pETM11 expression vector allowed hArg1 overexpressing in autoinduction media ZYM5052 [20] with a final yield of 40–60 mg hargI per liter culture after size exclusion chromatography. Human arginase II was sourced commercially from USBiological (Salem, USA) with a His6-tag at the N terminus.

### The synthetic peptide REGNH mimicking the hargI C-terminus binds to both wild-type hargI and hargII

**Fig. 2:**
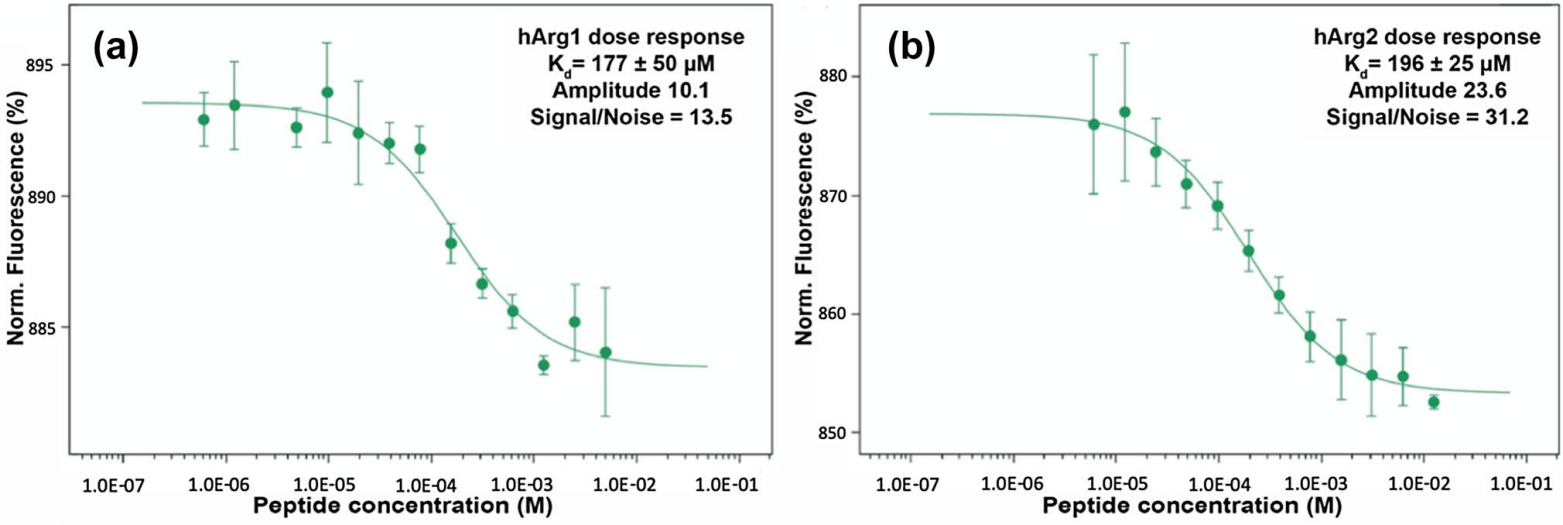
MST binding assay of synthetic peptide REGNH to hargI (**a**) and II (**b**), with serial peptide concentrations as indicated. The dose-response curve showed the interaction between the RED-tris-NTA dye labeled hargI/II and synthetic peptide analogue, each measurement was performed in triplicate. Fnorm = normalized fluorescence

The ability of the synthetic peptide analogue (REGNH) to bind to wild-type hargI and II was examined using Microscale Thermophoresis (MST) (Nanotemper, GmbH). In this experiment, fluorescently labeled hargI/II (see Materials and Methods) was incubated with serially diluted concentrations of the peptide REGNH. After 10 min incubation at room temperature the dose-response MST curve was measured, which yielded a clear binding curve with determined dissociation constant of 177 ± 50 μM for hargI, and 196 ± 25 μM for hargII (Fig. 2).

### The synthetic peptide REGNH inhibits hargI and II enzymatic activity

**Fig. 3:**
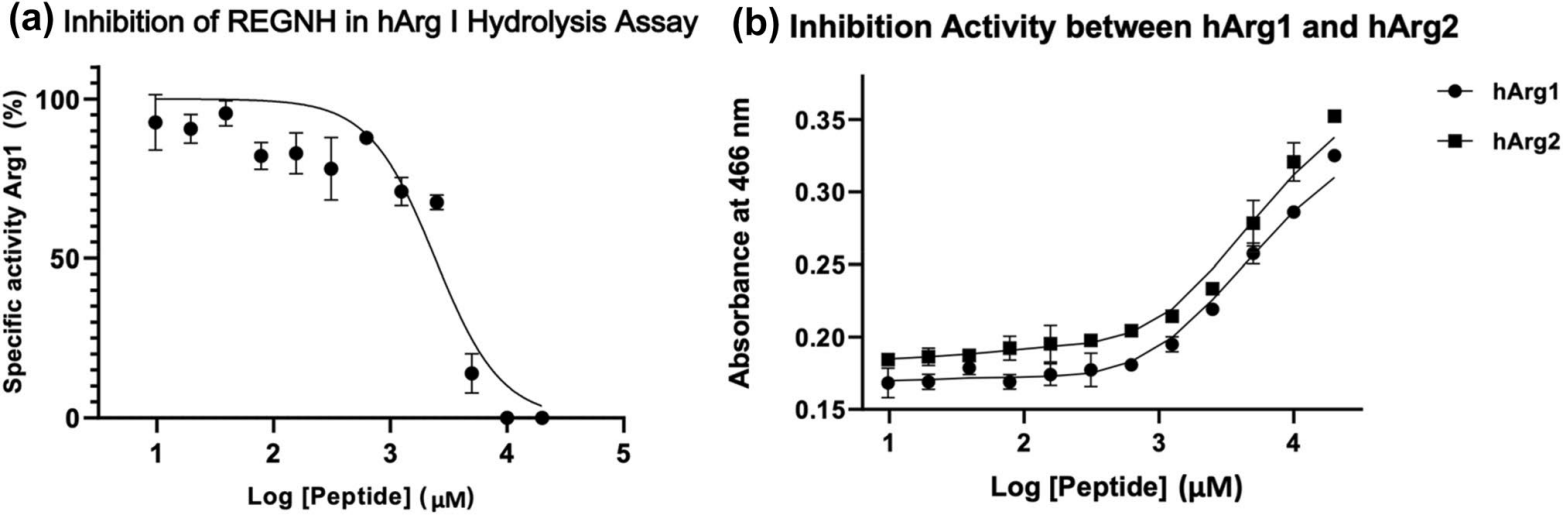
(**a**) Inhibition of the synthetic peptide REGNH against hargI followed in an enzymatic assay, measurements generated in triplicated. The relative enzyme activity was determined by measuring the hydrolysis product urea in a colorimetric assay, and the IC_50_ was calculated to be 2.4 ± 0.3 mM. (**b**) The inhibition activity of same synthetic peptide in L-arginine metabolism assay mediated by hargI and hargII, each OD measured in triplicate. The peptide REGNH concentration in both assays ranged from 9.8 μM to 20 mM. both inhibition enzymatic assay showed clear sigmoid curves with almost identical behavior

Enzymatic assays performed on full-length hargI showed that the peptide REGNH was able to inhibit hargI activity. Figure 3a showed a dose-response curve of hargI with an increasing peptide concentration (9.8 μM- 20 mM). The half maximal inhibitory concentration (IC_50_) was calculated as 2.4 ± 0.3 mM. Comparing with the MST result, which showed the binding ability of peptide to enzyme with a Kd ≈ 0.2 mM, the IC_50_ value was 12-fold larger than that measured by MST.

In order to assess the isoform specificity of the peptide REGNH, hargII was also screened under identical conditions. Based on the absorbance of hydrolyzed urea, we find that inhibition of the synthetic peptide against both enzymes are almost identical (Fig. 3b). The final IC_50_ of the peptide REGNH against hargII was determined to be 1.8 ± 0.1 mM, which is almost within experimental errors of hargI activity (2.4 mM).

### The Synthetic Peptide REGNH is not a competitive inhibitor

**Fig. 4:**
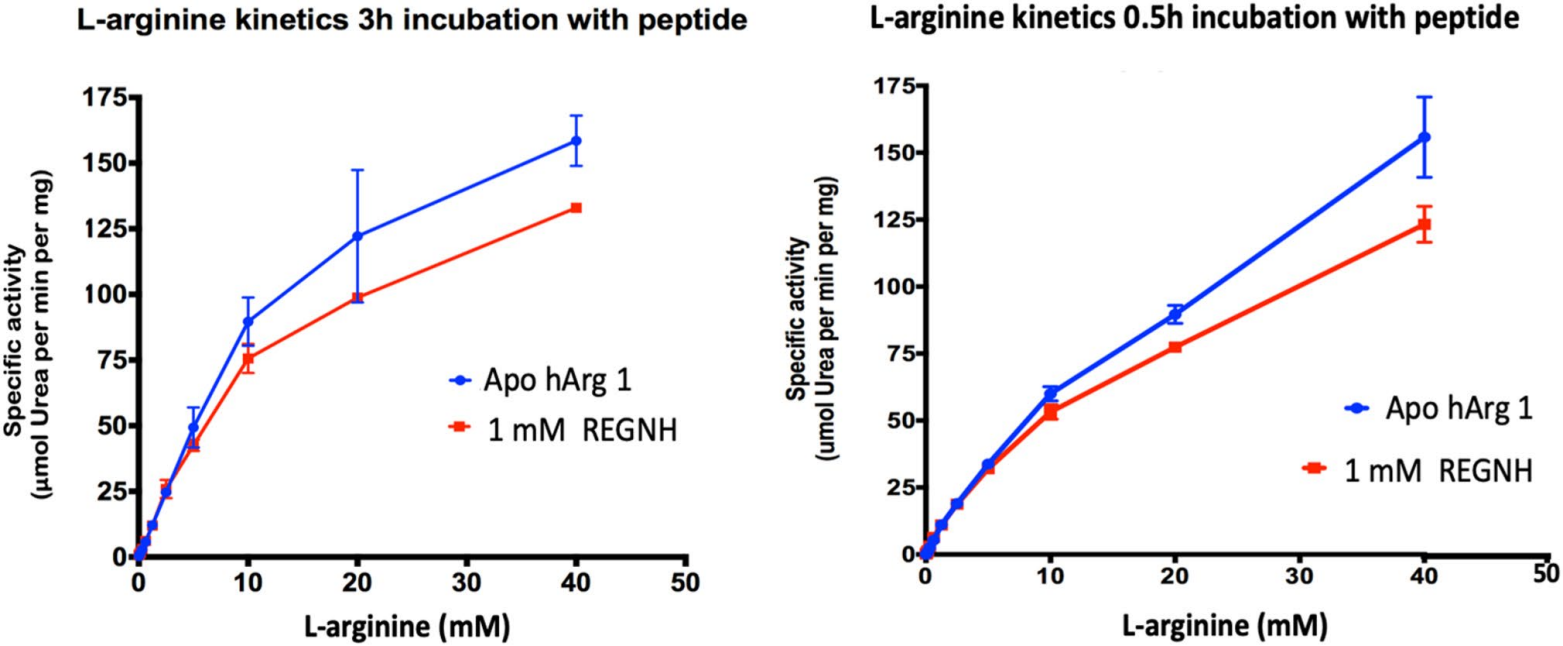
Kinetic assay of L-arginine metabolism in the absence/presence of peptide REGNH, measurements were taken after 0.5 h and 3 h and rate of urea production indicates the extent of hydrolysis. As shown in the two time points, at 0.5 h, the difference compared with peptide started to be obvious at 20 mM L-arginine, whereas it started at 10 mM at 3 h. Longer time incubation slightly increased the amount of urea, but not the inhibition

Kinetic measurements with the substrate (L-arginine) concentrations ranging from 25 μM to 40 mM showed that addition of the peptide REGNH did not change the cooperative interaction with the substrate that is typical for human arginase I. However, the presence of the peptide lowered the specific activity of hargI, suggesting a non-competitive nature of the interaction, as even at highest tested L-arginine concentration (40 mM), the addition of 1 mM peptide REGNH lowered activity by ≈15% with respect to the apo-enzyme (Fig. 4).

**Fig. 5:**
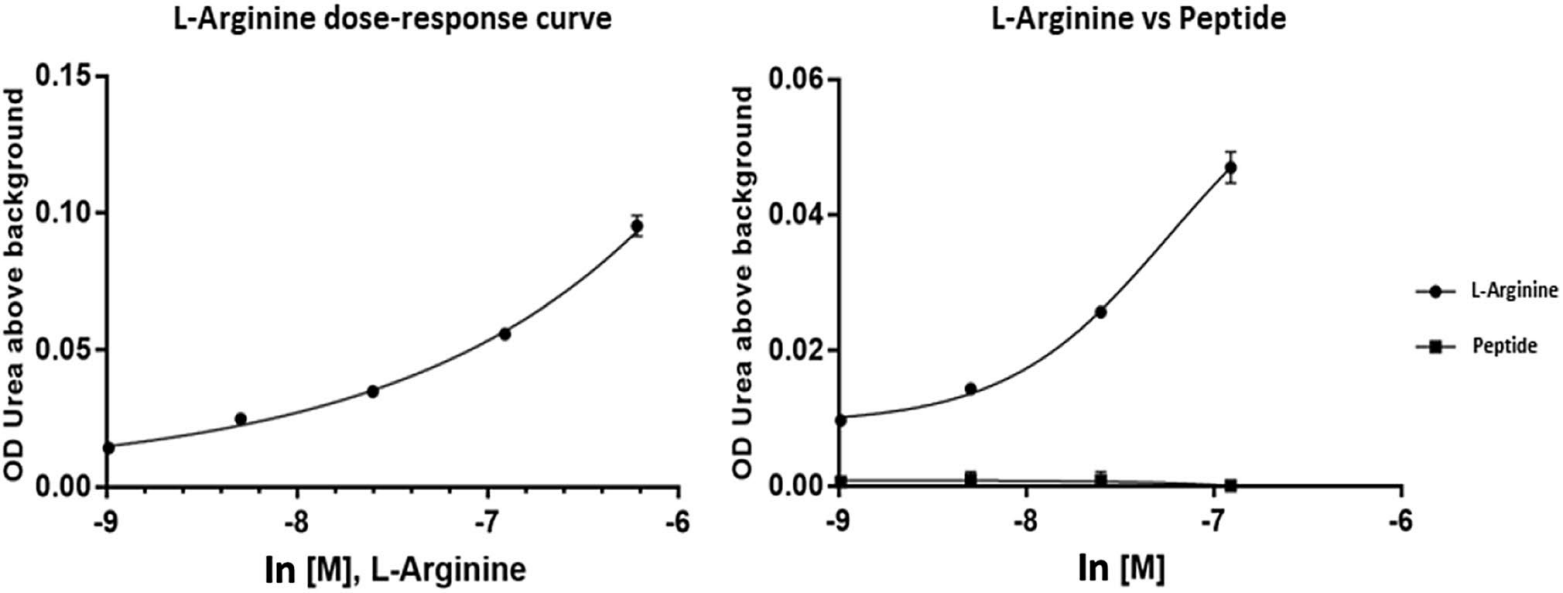
Comparison of L-arginine and peptide REGNH as substrates utilized by hargI, the curve on the upper right shows that the absorbance of peptide is negligible compared to L-arginine, indicating that the peptide was not converted to urea during the enzymatic assay

This hypothesis was further supported by a control experiment with the absence of L-arginine in the assay. As the peptide REGNH contains arginine in the sequence, the potential ability of hargI to use the peptide as substrate could not be excluded. In order to lower peptide consumption, the lowest amount of L-arginine necessary for the reliable measurement of urea production was assessed (Fig. 5), resulting in 1 mM substrate concentration for the following experiments. No production of urea could be detected in the absence of L-arginine, supporting the assumption that the REGNH C-terminus analogue peptide is not catabolized by hargI and is therefore not interacting with the active site residues.

### Impact of the synthetic peptide REGNH on inhibition by ABH

**Fig. 6:**
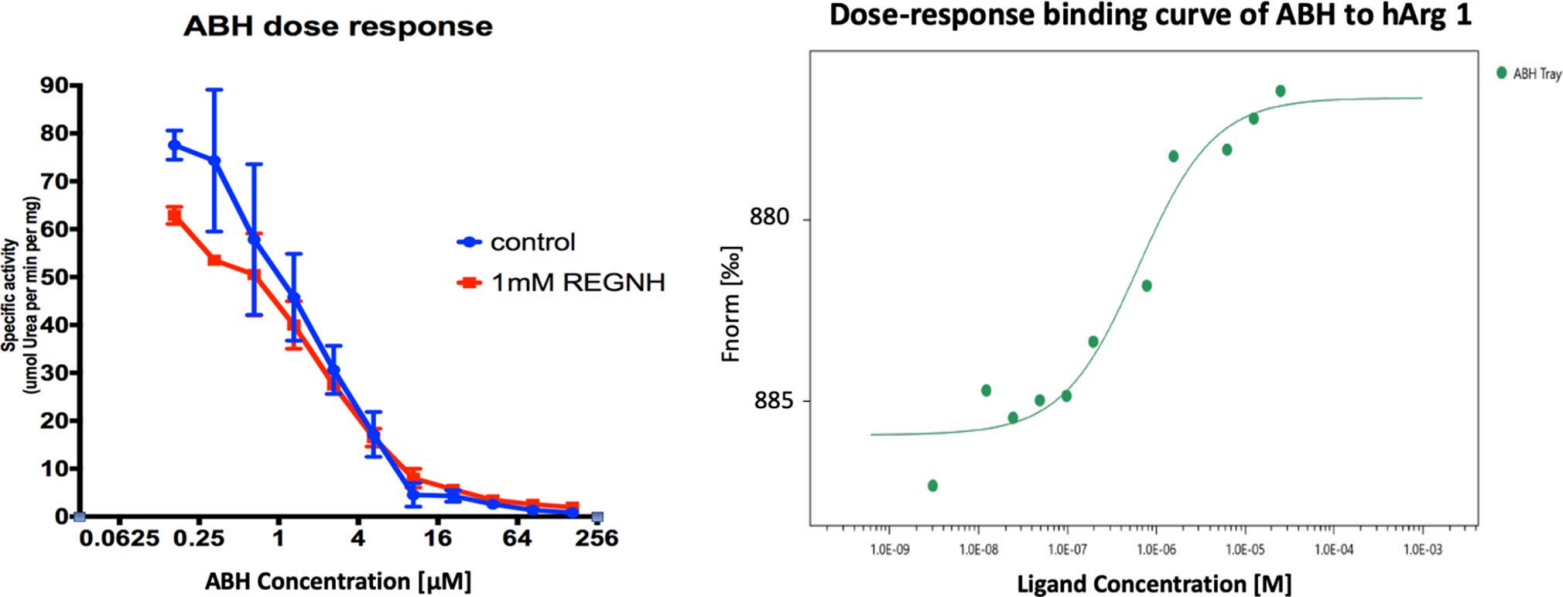
Effect of ABH as the known inhibitor in the presence/absence of synthetic peptide REGNH in the L-arginine metabolism (lower left). The dose-dependent MST assay showed a strong binding of ABH to harg1 (lower right)

In order to examine whether the presence of peptide REGNH affects the binding of the known hargI active site inhibitor 2-(S)-amino-6-boronohexanoic acid (ABH) [11], the following experiments were performed. ABH was synthesized in-house as described before [21]. The binding affinity of ABH was assessed by MST, showing a measured K_d_ of approximately 600 nM (Fig. 6). Although hargI containing 1 mM REGNH showed decreased activity, the analysis dose-response of hargI (supplemented with 1 mM peptide REGNH) to ABH showed comparable inhibition constants to those of the apo-enzyme (IC_50_ of 1 ± 0.03 μM). These data strongly suggest that REGNH peptide has a different inhibition mode and does not interfere with ABH mediated inhibition of hargI/II.

## Discussion

The aim of the described research is to examine the effect of the peptide analogue REGNH (residue 308–312), which comprises part of the S-shape motif from the C-terminal (residue 308–322) on hargI. A previous study has shown that the C terminal tail plays a critical role in retaining trimeric protein assembly, as well as in the cooperative response of arginase in L-arginine metabolism. Furthermore, compared to wild-type hargI, the activity of monomeric Arg308Ala mutant was significantly increased in the presence of low concentration Gdn^+^, where Gdn^+^ is suggested to mimic the guanidine group of Arg308 [17]. These discoveries show the essential role of Arg308 together with the S-shape motif in arginase activity, and it have been validated in the *Bacillus caldovelox* arginase co-crystal structure with guanidinium chloride and L-arginine [19].

The dramatic activity difference of truncated and mutated variants of hargI suggested that a designed short peptide that mimics the C terminal—including Arg308—could be an effective strategy in affecting function and may allow the development of species specific isoforms, based on interactions with divergent residues distant from the conserved active site. Our peptide REGNH does show inhibition activity in the mmol/L range in a L-arginine hydrolysis assay mediated by arginases, and the result corresponds with the findings reported by Garcia, in which 30% activity was lost by removing the last 14 amino acids (residues 308–322) from hargI. Further tests also validated that the analogue showed inhibition of both hargI and hargII in vitro assay, with the similar IC50 indicating the current peptide can inhibit L-arginine metabolism by arginases I and II without apparent distinctions. To date, no inhibitors reported selectivity between the two isoforms in enzymatic assays, this may be due to the identical Mn^2+^ cluster and active site configurations. In the strictly conserved active sites of the human arginase I [22] and arginase II [23] crystal structures, the guanidinium group of L-arginine forms a tetrahedral intermediate after the nucleophilic attack of metal-bridging hydroxide, and is subsequently degraded to L-ornithine and urea. However, selectivity of inhibitors to hargI and II is still desirable, as the two isoforms are distributed in different organs and cells, and specific treatment is required for certain diseases association with the different arginases.

The designed peptide shows non-competitive inhibition behavior in arginine hydrolysis, which implies that the interaction site is different from the substrate binding pocket. This was further confirmed in the assay performed using a known arginases inhibitor ABH, which competes with arginine at the Mn2+ activity site. The addition of REGNH does not change the inhibitor strength, but only lower the activity. For a SAR (Structure Activity Relationship) study and drug candidate optimization, the binding mode plays a key role in enzyme hydrolysis procedures, so how the peptide interact with human arginases still needs to be determined. Structure biology could provide more information for this EI (enzyme-inhibitor) or ESI (enzyme-substrate-inhibitor) complex binding mode, and X-ray diffraction or NMR are be two good options for this study.

Finally, the peptidomimetics derived from the partial sequence of the protein target could serve as an inhibitor for biological activity, and this strategy has been successful in many drug discovery projects [24, 25]. A recent important case points to the discovery and development of Liraglutide, a peptide analogue derived from residue7–37 of glucagon-like peptide-1(GLP-1) used to treat type 2 diabetes (T2D) and obesity since 2009 [26]. However, to fully optimize the property of peptide analogue as a drug in clinical usage, further investigations are still needed as transcellular uptake and enzymatic degradation remain the main traps before the peptides accomplish the desired function.

## Supplementary Information

Below is the link to the electronic supplementary material.

Electronic supplementary material 1 (DOCX 17 kb)

## References

[1] Wu G, Morris SM (1998). Arginine metabolism and nitric oxide production. Biochem J.

[2] Morris SM (2009). Recent advances in arginine metabolism: roles and regulation of the arginases. Br J Pharmacol.

[3] Pegg AE (2014). The function of spermine. IUBMB Life.

[4] Maarsingh H, Zuidhof AB, Bos IST, Van Duin M, Boucher JL, Zaagsma J, Meurs H (2008). Arginase inhibition protects against allergen-induced airway obstruction, hyperresponsiveness, and inflammation. Am J Respir Crit Care Med.

[5] Jenkinson CP, Grody WW, Cederbaum SD (1996). Comparative properties of arginases. Comp Biochem Physiol – B Biochem Mol Biol.

[6] Mori M, Gotoh T (2004). Arginine metabolic enzymes, nitric oxide and infection. J Nutr.

[7] Ruth BC, Haroldo AT, Priya N, William C (2015). Arginase: an old enzyme with new tricks. Trends Pharmacol Sci.

[8] Patil M, Bhaumik J, Babykutty S, Banerjee U, Fukumura D (2016). Arginine dependence of tumor cells: targeting a chink in cancer’s armor. Oncogene.

[9] Pudlo M, Demougeot C, Girard-Thernier C (2017). Arginase inhibitors: a rational approach over one century. Med Res Rev.

[10] Boucher JL, Custot J, Vadon S, Delaforge M, Lepoivre M, Tenu JP, Yapo A, Mansuy D (1994). Nω-Hydroxy-L-arginine, an intermediate in the L-arginine to nitric oxide pathway, is a strong inhibitor of liver and macrophage arginase. Biochem Biophys Res Commun.

[11] Baggio R, Elbaum D, Kanyo ZF, Carroll PJ, Cavalli RC, Ash DE, Christianson DW (1997). Inhibition of Mn2+2-arginase by borate leads to the design of a transition state analogue inhibitor, 2(S)-amino-6-boronohexanoic acid. J Am Chem Soc.

[12] Kim NN, Cox JD, Baggio RF, Emig FA, Mistry SK, Harper SL, Speicher DW, Morris SM, Ash DE, Traish A, Christianson DW (2001). Probing erectile function: S-(2-boronoethyl)-L-cysteine binds to arginase as a transition state analogue and enhances smooth muscle relaxation in human penile corpus cavernosum. Biochemistry.

[13] Grobben Y, Uitdehaag JCM, Willemsen-Seegers N, Tabak WWA, Man J, Buijsman RC, Zaman GJR (2019). Structural insights into human Arginase-1 pH dependence and its inhibition by the small molecule inhibitor CB-1158. J Struct Biol: X.

[14] Mora A, del Ara RM, Fuentes JM, Soler G, Centeno F (2000). Implications of the S-shaped domain in the quaternary structure of human arginase. Biochim Biophys Acta – Protein Struct Mol Enzymol.

[15] Lavulo LT, Sossong TM, Brigham-Burke MR, Doyle ML, Cox JD, Christianson DW, Ash DE (2001). Subunit-subunit interactions in trimeric arginase. Generation of active monomers by mutation of a single amino acid. J Biol Chem.

[16] Dowling DP, Di Costanzo L, Gennadios HA, Christianson DW (2008). Evolution of the arginase fold and functional diversity. Cell Mol Life Sci.

[17] García D, Uribe E, Lobos M, Orellana MS, Carvajal N (2009). Studies on the functional significance of a C-terminal S-shaped motif in human arginase type I: essentiality for cooperative effects. Arch Biochem Biophys.

[18] N. Carvajal, M. Acoria, J.P. Rodríguez, M. Fernández, J. Martínez, J (1982) Evidence for cooperative effects in human liver arginase. Biochim Biophys Acta 701, 146–148 10.1016/0167-4838(82)90324-7 7055584

[19] Bewley MC, Jeffrey PD, Patchett ML, Kanyo ZF, Baker EN (1999). Crystal structures of Bacillus caldovelox arginase in complex with substrate and inhibitors reveal new insights into activation, inhibition and catalysis in the arginase superfamily. Structure.

[20] Studier FW (2005). Protein production by auto-induction in high density shaking cultures. Protein Expr Purif.

[21] Zhao T (2016) Novel applications of Tetrazoles derived from the TMSN3-Ugi reaction

[22] Di Costanzo L, Sabio G, Mora A, Rodriguez PC, Ochoa AC, Centeno F, Christianson DW (2005). Crystal structure of human arginase I at 1.29-Å resolution and exploration of inhibition in the immune response. PNAS.

[23] Cox N, Baggio JD, Emig RF, Mistry FA, Harper S, Speicher SL, Morris DW, Ash SM, Traish DE, Christianson AM (2003). Human arginase II: crystal structure and physiological role in male and female sexual arousal. Biochemistry.

[24] Wang X, Qiao Y, Asangani IA, Ateeq B, Poliakov A, Cieślik M, Pitchiaya S, Chakravarthi BVSK, Cao X, Jing X, Wang CX, Apel IJ, Wang R, Tien JCY, Juckette KM, Yan W, Jiang H, Wang S, Varambally S, Chinnaiyan AM (2017). Development of Peptidomimetic inhibitors of the ERG gene fusion product in prostate Cancer. Cancer Cell.

[25] Qvit N, Rubin SJS, Urban TJ, Mochly-Rosen D, Gross ER (2017). Peptidomimetic therapeutics: scientific approaches and opportunities. Drug Discov Today.

[26] Knudsen LB, Lau J (2019). The discovery and development of liraglutide and semaglutide. Front Endocrinol (Lausanne).

[27] Ericsson UB, Hallberg BM, DeTitta GT, Dekker N, Nordlund P (2006). Thermofluor-based high-throughput stability optimization of proteins for structural studies. Anal Biochem.

[28] Prescott LM, Jones ME (1969). Modified methods for the determination of carbamyl aspartate. Anal Biochem.

[29] Golebiowski A, Paul Beckett R, Van Zandt M, Ji MK, Whitehouse D, Ryder TR, Jagdmann E, Andreoli M, Mazur A, Padmanilayam M, Cousido-Siah A, Mitschler A, Ruiz FX, Podjarny A, Schroeter H (2013). 2-Substituted-2-amino-6-boronohexanoic acids as arginase inhibitors. Bioorganic Med Chem Lett.

[30] Van Zandt MC, Whitehouse DL, Golebiowski A, Ji MK, Zhang M, Beckett RP, Jagdmann GE, Ryder TR, Sheeler R, Andreoli M, Conway B, Mahboubi K, D’Angelo G, Mitschler A, Cousido-Siah A, Ruiz FX, Howard EI, Podjarny AD, Schroeter H (2013). Discovery of (R)-2-amino-6-borono-2-(2-(piperidin-1-yl)ethyl) hexanoic acid and congeners as highly potent inhibitors of human arginases i and II for treatment of myocardial reperfusion injury. J Med Chem.

