# Supplementary material for: A synthetic peptide as an allosteric inhibitor of human arginase I and II
Source: Mol Biol Rep. 2021 Feb 15;48(2):1959–66. doi: 10.1007/s11033-021-06176-5 (PMC7925462; doi:10.1007/s11033-021-06176-5)
Supplement: Supplementary file 1 — Electronic supplementary material 1 (DOCX 17 kb) [file 11033_2021_6176_MOESM1_ESM.docx]

Supporting Information: hArg1 cloning details

| Source organism | *Homo sapiens* |
| --- | --- |
| DNA source | Plasmid repository (dnasu.org) |
| Gene name | hArg1 |
| PCR-amplified sequence | ATGAGCGCCA AGTCCAGAAC CATAGGGATT ATTGGAGCTC CTTTCTCAAA GGGACAGCCA  CGAGGAGGGG TGGAAGAAGG CCCTACAGTA TTGAGAAAGG CTGGTCTGCT TGAGAAACTT  AAAGAACAAG AGTGTGATGT GAAGGATTAT GGGGACCTGC CCTTTGCTGA CATCCCTAAT  GACAGTCCCT TTCAAATTGT GAAGAATCCA AGGTCTGTGG GAAAAGCAAG CGAGCAGCTG  CTGGCAAGGT GGCAGAAGTC AAGAAGAACG GAAGAATCAG CCTGGTGCTG GGCGGAGACC ACAGTTTGGC AATTGGAAGC ATCTCTGGCC ATGCCAGGGT CCACCCTGAT CTTGGAGTCA TCTGGGTGGA TGCTCACACT GATATCAACA CTCCACTGAC AACCACAAGT GGAAACTTGC ATGGACAACC TGTATCTTTC CTCCTGAAGG AACTAAAAGG AAAGATTCCC GATGTGCCAG GATTCTCCTG GGTGACTCCC TGTATATCTG CCAAGGATAT TGTGTATATT GGCTTGAGAG ACGTGGACCC TGGGGAACAC TACATTTTGA AAACTCTAGG CATTAAATAC TTTTCAATGA CTGAAGTGGA CAGACTAGGA ATTGGCAAGG TGATGGAAGA AACACTCAGC TATCTACTAG GAAGAAAGAA AAGGCCAATT CATCTAAGTT TTGATGTTGA CGGACTGGAC CCATCTTTCA CACCAGCTAC TGGCACACCA GTCGTGGGAG GTCTGACATA CAGAGAAGGT CTCTACATCA CAGAAGAAAT CTACAAAACA GGGCTACTCT CAGGATTAGA TATAATGGAA GTGAACCCAT CCCTGGGGAA GACACCAGAA GAAGTAACTC GAACAGTGAA CACAGCAGTT GCAATAACCT TGGCTTGTTT CGGACTTGCT CGGGAGGGTA ATCACAAGCC TATTGACTAC CTTAACCCAC CTAAG |
| Forward primer | 5'-GGCGGC**TCATGA**GCGCCAAGTCCAGAACCATA-3' |
| Reverse primer | 5'-GCCGCC**AAGCTT**ACTTAGGTGGGTTAAGGTAGTCAATA-3' |
| Cloning vector | pANT7_cGST_ARG1 |
| Expression vector | pETM-11 |
| Expression host | *Escherichia coli* |
| Complete amino acid sequence of construct (including the expression tag and linker sequence) | MK**HHHHHH**PMSDYDIPTTENLYFEGAMSAKSRTIGIIGAPFSKGQPRGGVEEGPTV  LRKAGLLEKLKEQECDVKDYGDLPFADIPNDSPFQIVKNPRSVGKASEQLAGKVAE  VKKNGRISLVLGGDHSLAIGSISGHARVHPDLGVIWVDAHTDINTPLTTTSGNLHG  QPVSFLLKELKGKIPDVPGFSWVTPCISAKDIVYIGLRDVDPGEHYILKTLGIKYF  SMTEVDRLGIGKVMEETLSYLLGRKKRPIHLSFDVDGLDPSFTPATGTPVVGGLTY  REGLYITEEIYKTGLLSGLDIMEVNPSLGKTPEEVTRTVNTAVAITLACFGLAREG  NHKPIDYLNPPK |
